# One-pot synthesis for gradient copolymers *via* concurrent tandem living radical polymerization: mild and selective transesterification of methyl acrylate through Al(acac)_3_ with common alcohols†

**DOI:** 10.1039/d1ra04595d

**Published:** 2021-07-28

**Authors:** Tam Thi-Thanh Huynh, Si Eun Kim, Soon Cheon Kim, Jin Chul Kim, Young Il Park, Ji-Eun Jeong, Hyeonuk Yeo, Sang-Ho Lee

**Affiliations:** Center for Advanced Specialty Chemicals, Korea Research Institute of Chemical Technology Ulsan 44412 Republic of Korea; Department of Science Education, Kyungpook National University Daegu 41566 Republic of Korea; Department of Chemistry Education, Department of Pharmacy, Kyungpook National University Daegu 41566 Republic of Korea slee@krict.re.kr yeo@knu.ac.kr

## Abstract

A series of gradient copolymers were synthesized by the ruthenium-catalyzed living radical polymerization (LRP) of methyl acrylate (MA) and aliphatic alcohols using aluminum acetylacetonate Al(acac)_3_. In this polymerization system, Al(acac)_3_ was successfully used not only as an additive for the Ru-catalyzed LRP but also as a catalyst for the selective transesterification of an unsaturated ester monomer in mild conditions in a process known as concurrent tandem living radical polymerization. The resulting MA-based gradient copolymers showed well-controlled molecular weight and distribution in a one-pot reaction and exhibited a well-controlled gradient sequence in their polymer chain. Control of transesterification and the metal-catalyzed living radical polymerization (Mt-LRP) rate varied depending on the concentration of the Al(acac)_3_ and the structure of varying alcohols, which were confirmed by ^1^H NMR, SEC, and DSC analysis. In particular, this research opens a new synthetic methodology for preparing acrylate-based gradient copolymers *via* concurrent tandem LRP not limited to the synthesis of methyl methacrylate types of gradient copolymers.

## Abstract 2

The acrylate based gradient copolymers were successfully synthesized by concurrent tandem living radical polymerization using Al(acac)_3_ as cocatalyst with common alcohols.

## Graphical abstract

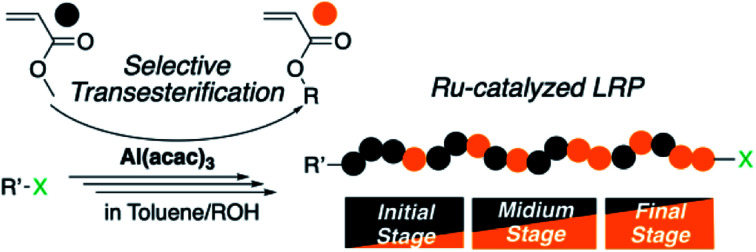

## Introduction

After the first proposal for the concept of a “gradient copolymer” in 1977,^1^ the studies of their synthesis and properties have attracted significant interest in related fields. Gradient copolymers possess highly interesting molecular structures, in which a progressive transition of repeating components continues along the development of a polymer chain. Owing to the continuously changing structural nature along the chains, the gradient copolymers have unique properties which lead to less intra- and interchain repulsion, unlike block copolymers. These characteristics make them usable in a number of applications such as in thermoplastic elastomers,^2^ nanostructured carbons,^3^ multishape memory materials,^4^ dispersants, functionalized surfaces, bio-medical materials,^5,6^ and vibration damping materials.^7^

In the past decades, the living radical polymerization (LRP) method has been developed as a powerful tool which has significantly impacted the field of polymer materials synthesis. Specifically, nitroxide-mediated radical polymerization (NMP),^8,9^ reversible addition–fragmentation chain transfer radical polymerization (RAFT),^10–12^ and metal-catalyzed living radical polymerization (Mt-LRP)^13–16^ have been used to synthesize gradient copolymers. Among these, Mt-LRP is known as a prominent method to synthesis various polymers with tailoring polydispersity and molecular weight distribution such as homopolymers, block copolymers, random copolymers, and star polymers.^17–19^ In addition, Mt-LRP systems have attracted much attention based on high controllability in the addition of metal alkoxides, which not only control the molecular weights but also increase the polymerization rate.

Over the past few years, Sawamoto and Terashima have reported a new synthetic strategy for the preparation of gradient copolymers in one-pot synthesis using a concurrent catalysis system.^20–22^ This polymerization system, known as concurrent tandem LRP, is a combination of the transesterification of an unsaturated ester monomer with alcohols using a metal alkoxide such as Al(O*i*-Pr)_3_ or Ti(O*i*-Pr)_4_ and ruthenium (Ru)-catalyzed LRP in conjunction with a metal alkoxide as a cocatalyst, and has led to well-defined polymer structures *via* resulting smooth redox reactions.^13^ Thereby, it induces selective transesterification for only the methyl methacrylate monomer (MMA) type monomer with various alcohols (not polymer chain in propagation), which allows for well-controlled gradient sequences in the polymer chain. However, this strategy is not perfectly applicable to the acrylate type monomer owing to the high reactivity of metal alkoxide, which led to partial transesterification of the polymer chains.

**Scheme 1:**
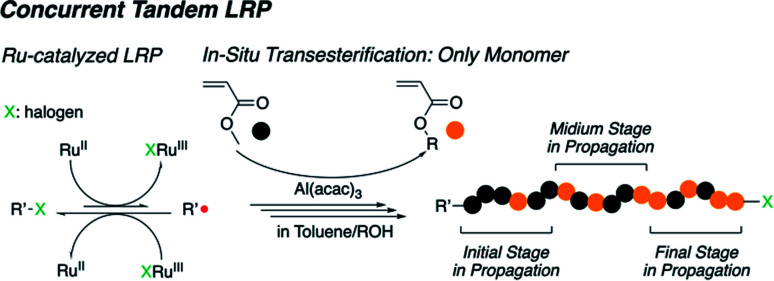
Concurrent tandem living radical polymerization for acrylate-based gradient copolymer synthesis.

Thus, in this work, we report the concurrent tandem LRP used to prepare an acrylate-based gradient copolymer using aluminum (Al) acetylacetonate (Al(acac)_3_). The Al(acac)_3_ is also an effective catalyst for transesterification and has relatively lower Lewis acidity than other metal alkoxides.^21^ In addition, it can be used as an additive to Ru-catalyzed LRP.^13^ These two roles of Al(acac)_3_ for each reaction are successfully synchronized in the concurrent tandem LRP to create an acrylate-based gradient copolymer carrying a well-controlled gradient sequence in the polymer chain in a one-pot synthesis through the gradual transesterification of the acrylate monomer (Scheme 1). Note that our focus is to expand the monomer scope in concurrent tandem LRP as well as synthesis of well-defined gradient copolymers.

## Experimental section

### Materials

Methyl acrylate (MA, Sigma-Aldrich, 99%) was purified before use by a neutral aluminum oxide column to remove the inhibitor and was kept at 0 °C. Ru(Cp*)Cl(PPh_3_)_2_ (Sigma-Aldrich), tetralin (Sigma-Aldrich, 99%), and ethyl 2-bromopropionate (EBP, Sigma-Aldrich, purity >98%) were used as received and kept at 0 °C. Al(acac)_3_ (Sigma-Aldrich, purity >97%), ethanol (Sigma-Aldrich, anhydrous, ≥99.5%), iso-propanol (Sigma-Aldrich, anhydrous, ≥99.5%), octanol (Sigma-Aldrich, ACS reagent, ≥99%), and benzyl alcohol (Sigma-Aldrich, anhydrous, 99.8%) were degassed before use. Toluene (Sigma-Aldrich; ≥99.9%) was purified by being passed through purification columns (JCM, JCM-3SPS-SA-6) and bubbled with dry nitrogen gas for more than 15 min immediately before use. Molecular sieves 4 Å (MS 4 Å) were dried under reduced pressure at 300 °C by a heat gun before use.

### Measurements

The *M*_n_ and *M*_w_/*M*_n_ of polymers were measured by SEC at 40 °C using tetrahydrofuran (THF) as an eluent. For the THF-SEC, three polystyrene-gel columns [GP LF-404 (from Shodex); pore size, 3000 Å; 4.6 mm i. d. × 250 mm]; were connected to a PU-4180 pump, a RI-4030 refractive-index detector, and a UV-4075 ultraviolet detector (JASCO) and the flow rate was set to 0.3 mL min^−1^. The columns were calibrated against 13 standard poly(methyl methacrylate) samples (Agilent Technologies; Mp = 2380–1120 000). Proton nuclear magnetic resonance (^1^H NMR) was recorded on a Bruker Ultrashield spectrometer operating at 300 MHz. The thermal properties of the gradient copolymer were determined using differential scanning calorimetry (DSC, TA 3000).

### Al(acac)_3_-catalyzed transesterification of MA with varying alcohols

Transesterification was conducted using the syringe technique under dry Ar in baked glass tubes equipped with a three-way stopcock. A typical procedure for the MA with varying alcohols is given. For example, MS 4 Å (0.33 g mL^−1^) was added into a tube and dried *in vacuo*. In another 30 mL tube, toluene (1.46 mL), ethanol (1.96 mL), tetralin (0.14 mL), a 0.2 M toluene solution of Al(acac)_3_ (0.5 mL, 0.1 mmol), and MA (10 mmol, 0.95 mL) were added in the same order at room temperature. The solution was added into to the glass tube containing MS 4 Å. The total volume of the reaction mixture was controlled to 5 mL. Then, the mixture was immediately moved in an oil bath that was at 80 °C. Aliquots from the reaction solution were withdrawn in predetermined intervals using a syringe and the subsequent reaction was terminated by cooling to −78 °C. The conversion of transesterification was determined using ^1^H NMR spectroscopy.

### Metal-catalyzed living radical polymerization of MA using Al(acac)_3_ as an additive

The Mt-LRP was conducted using a similar procedure described above and a typical procedure for MA with varying alcohols is given. Ru(Cp*)Cl(PPh_3_)_2_ (0.0398 g, 0.05 mmol), toluene 3.66 mL, tetralin (0.14 mL), MA (0.95 mL, 10 mmol), and EBP (0.25 mL of 0.4 M in toluene, 0.1 mol) were sequentially added into a 30 mL glass tube. The total volume of the reaction mixture was controlled to 5 mL and immediately heated to 80 °C. Aliquots from the reaction solution were withdrawn in predetermined intervals using a syringe, and the subsequent reaction was terminated by cooling to −78 °C. Total monomer conversion was determined using ^1^H NMR spectroscopy using an internal standard.

### Gradient copolymer synthesis *via* tandem catalyst

The gradient copolymerization with tandem catalyst was carried out by a similar procedure described above and a typical procedure for MA with varying alcohols is given. For example, MS 4 Å (0.33 g mL^−1^) was added into a tube and dried *in vacuo*. In another tube, Ru(Cp*)Cl(PPh_3_)_2_ (0.0398 g, 0.05 mmol), toluene (1.33 mL), tetralin (0.14 mL), Al(acac)_3_ (0.5 mL of 0.2 M in toluene, 0.1 mmol), MA (0.95 mL, 10 mmol), EtOH (1.83 mL), and EBP (0.25 mL of 0.4 M in toluene, 0.1 mmol) were sequentially added. The solution was added into to the glass tube containing MS 4 Å and the total volume was controlled to 5 mL. The mixture was immediately heated to 80 °C and aliquots from the reaction solution were withdrawn in predetermined intervals using a syringe. The polymerization was terminated by cooling to −78 °C. Total monomer conversion and monomer compositions in polymer solution were determined using ^1^H NMR spectroscopy using an internal standard. The products in each aliquot were obtained upon solvent removal using rotary evaporation and further dried under reduced pressure. The cumulative (*F*_cum_) contents and instantaneous (*F*_inst_) contents were calculated from the repeat-unit composition of resulting polymers, which was determined using ^1^H NMR spectroscopy.

### Al(acac)_3_-catalyzed transesterification of poly(methyl acrylate) (PMA) with ethanol

The transesterification of PMA_100_ was conducted using a similar procedure as described above. MS 4 Å (0.33 g mL^−1^) was added into a tube and dried *in vacuo*. In another tube, 0.88 g of PMA_100_ (*M*_n,SEC_ = 10 200, *M*_w_/*M*_n_ = 1.16, DP_n,NMR_ = 100), toluene (1.93 mL), EtOH (2.43 mL), tetralin (0.14 mL), and a 0.2 M toluene solution of Al(acac)_3_ (0.5 mL, 0.1 mmol) were sequentially added. The solution was added into to the glass tube containing MS 4 Å and the total volume was controlled to 5 mL. The mixture was immediately heated to 80 °C and aliquots from the reaction solution were withdrawn in predetermined intervals using a syringe. The reaction was terminated by cooling to −78 °C and the products in each aliquot were obtained upon solvent removal using rotary evaporation. The product was further dried under reduced pressure and the structural change was tracked using ^1^H NMR spectroscopy.

## Results and discussion

### Al(acac)_3_-catalyzed transesterification of MA and PMA with alcohols

**Fig. 1:**
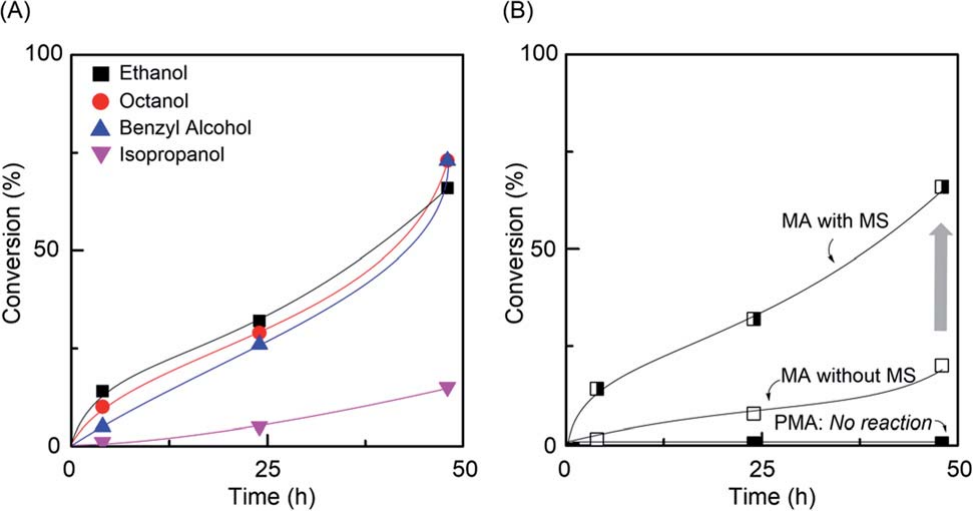
Al(acac)_3_-catalyzed transesterification of (A) MA with varying alcohols and (B) PMA with ethanol in toluene at 80 °C: [MA]_0_ = 2.0 M or [PMA (DP_n,NMR_ = 100)]_0_ = 20 mM; [Al(acac)_3_]_0_ = 20 mM in toluene/alcohols (1/1, v/v).

To obtain the well-controlled gradual composition of the monomer used in the gradient copolymer chain by *in situ* transesterification, the following factors had to be satisfied: (i) transesterification should have selectively occurred to the monomer only (not the polymer); (ii) the cooperative catalytic system was required for the Ru-catalyzed LRP and transesterification; (iii) the propagation must have been living without termination and chain transfer.^20,22^ Thus, Al(acac)_3_ was used to the transesterification of MA and PMA in alcohol/toluene (1/1, v/v) at 80 °C (Fig. 1). The transesterification of MA was observed for different alcohols, but PMA (*M*_n,SEC_ = 10 200, *M*_w_/*M*_n_ = 1.16, DP_n,NMR_ = 100) did not participate in the transesterification reaction, indicating that the selective transesterification occurred (Fig. S1 and S2†). Particularly, primary alcohols showed higher transesterification efficiency than secondary alcohol due to its higher reactivity. In addition, in molecular sieve (MS, 4 Å), the transesterification efficiency was enhanced by removing of the methanol molecule that formed during the reaction.^23^

### Effect of Al(acac)_3_ as an additive for Ru-catalyzed LRP

The polymerization of MA was performed with varying initiators such as EBP, EBiB, and ECPA. Three Ru complexes, Ru(Cp*)Cl(PPh_3_)_2_ (RuCp*),^24^ RuCl_2_(PPh_3_)_2_ (RuPPh),^25^ and Ru(Ind)Cl(PPh_3_)_2_ (RuInd)^26^ were examined as catalysts with Al(acac)_3_ as an additive (Table 1). With the EBP initiator, RuCp* gave controlled molecular weight, quantitative initiation, and narrower molecular weight distribution (MWD) (*M*_w_/*M*_n_ < 1.1: entry 1 in Table 1), and this polymerization system was based on the optimization of the reaction conditions for polymer preparation.

**Table 1:** Ru-catalyzed LRP of MA with various initiators in conjunction with Al(acac)_3_ as an additive^a^

Entry	Initiator	Catalyst	Time (h)	Conv.^b^ (%)	*M*_n_ c	*M*_w_/*M*_n_^c^
1	EBP	RuCp*	48	78	4900	1.09
2	EBP	RuPPh	96	65	6200	1.35
3	EBP	RuInd	24	35	1200	2.57
4	EBiB	RuPPh_3_	96	32	1000	1.58
5	EBiB	RuInd	96	54	4300	1.67
6	ECPA	RuPPh	96	53	4400	1.31
7	ECPA	RuInd	96	47	6800	1.92

a [MA]_0_ = 2.0 M; [initiator]_0_ = 20 mM; [Ru]_0_ = 2.0 mM; [Al(acac)_3_]_0_ = 10 mM in toluene at 80 °C.

b Conversion was determined by ^1^H NMR.

c Measured by size-exclusion chromatography calibrated with PMMA standards in THF (40 °C, flow rate 0.3 mL min^−1^).

**Fig. 2:**
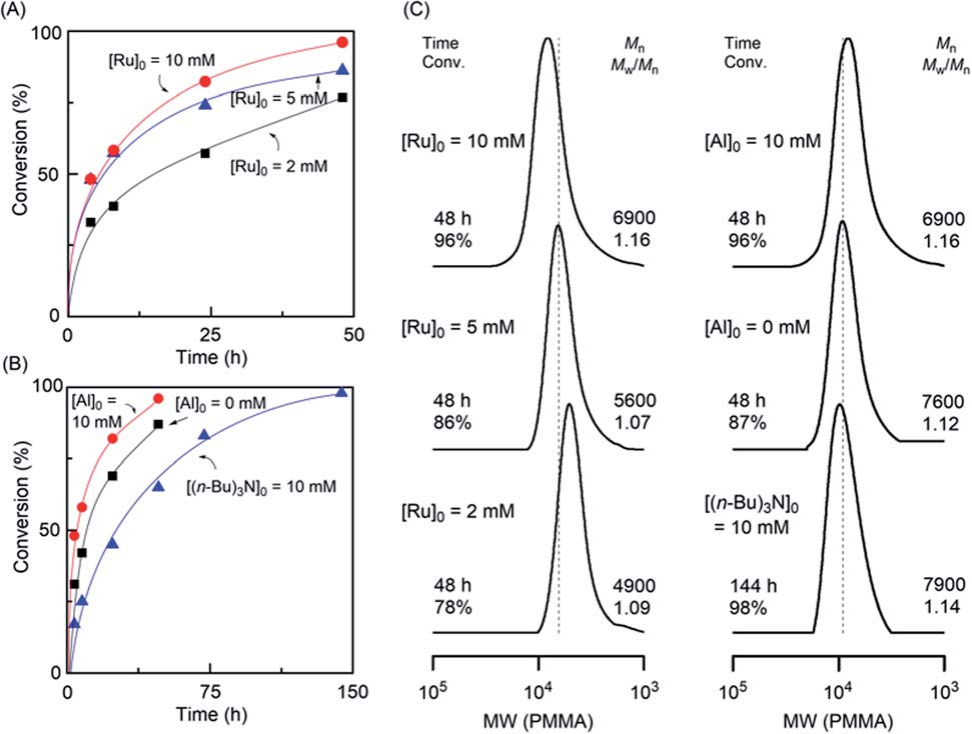
Homopolymerization of methyl acrylate with (A) varying ratios of Ru catalyst at a fixed ratio of Al cocatalyst, (B) varying cocatalyst concentration at a fixed ratio of Ru catalyst, and (C) corresponding SEC traces of the respective polymerizations with (left) the varying ratio of Ru catalyst at a fixed ratio of Al cocatalyst, and (right) varying cocatalyst at a fixed ratio of Ru catalyst. Dotted lines are included to aid the visual comparison. Reaction conditions: [MA]_0_ = 2.0 M; [EBP]_0_ = 20 mM; [Ru(Cp*)Cl(PPh_3_)_2_]_0_ = 2–10 mM; [additive]_0_ = 0–10 mM in toluene at 80 °C.

To optimize the catalyst amount in the polymerization conditions, varying concentrations of RuCp* from 2 to 10 mM were examined using 10 mM Al(acac)_3_ and MA was smoothly consumed up to a higher conversion. The higher concentration of RuCp* showed a fast polymerization rate with linearly increased molecular weight (*M*_n,SEC_) and controlled dispersity, suggesting a controlled nature of the LRP (Fig. 2A, C, S3, and S4†). Further, the effect of Al(acac)_3_ as an additive was evaluated compared with the absence of the Al cocatalyst ([Al(acac)_3_]_0_ = 0 mM) and the (*n*-Bu)_3_N cocatalyzed polymerization system (Fig. 2B and C). In Al cocatalyst, a fast reaction was afforded by keeping controlled MWDs similar to other polymerization conditions, indicating that Al(acac)_3_ can be considered not only a transesterification catalyst but also an additive for Ru-catalyzed LRP.

### Gradient copolymerization of MA with alcohols *via* concurrent tandem LRP

**Fig. 3:**
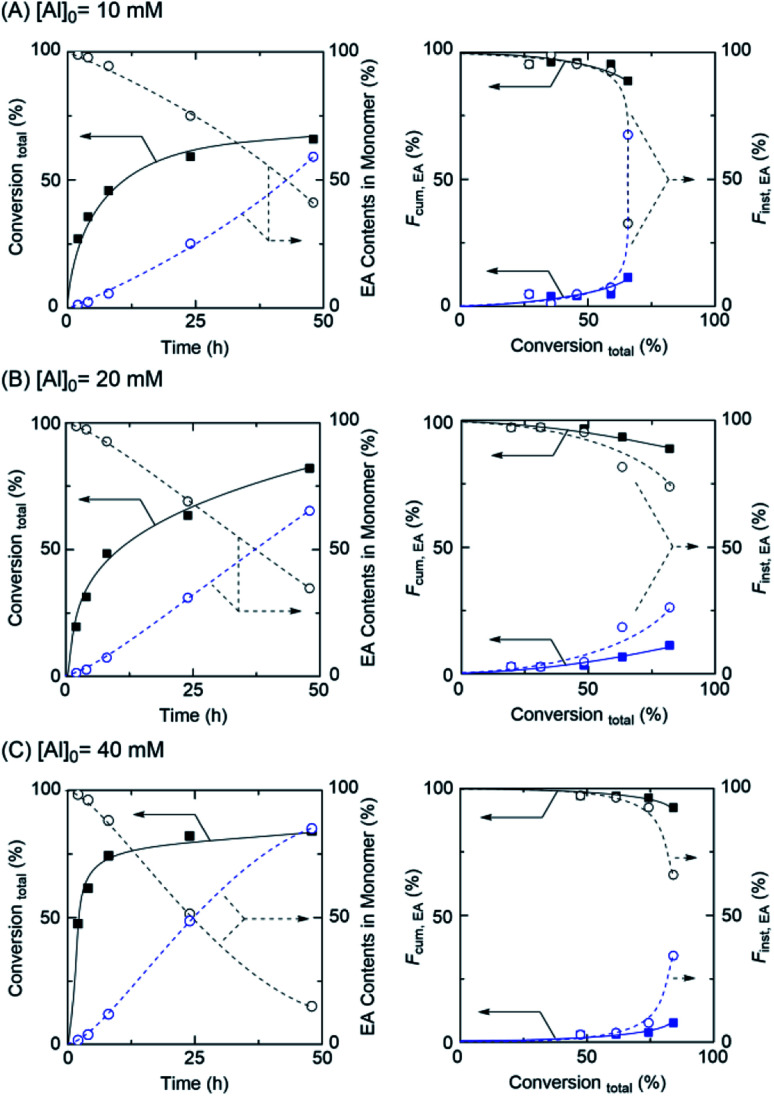
Effect of Al(acac)_3_ concentration on the synthesis of MA/EA gradient copolymer by concurrent tandem LRP: total conversion and EA contents in polymerization solution (left) and cumulative (*F*_cum,EA_) or instantaneous (*F*_inst,EA_) contents in polymer chains as a function of total conversion using (A) [Al]_0_ = 10 mM, (B) [Al]_0_ = 20 mM, and (C) [Al]_0_ = 40 mM: [MA]_0_ = 2.0 M; [EBP]_0_ = 20 mM; [Ru(Cp*)Cl(PPh_3_)_2_]_0_ = 10 mM in toluene/ethanol (1/1, v/v) at 80 °C.

Based on the discussed above, to control the gradient sequence in a polymer chain by concurrent tandem LRP, Al(acac)_3_ was used for acrylate-based gradient copolymer synthesis with ethanol. As demonstrated in Fig. 3 (left), for example, the polymerization smoothly proceeded up to higher conversion and ethyl acrylate content (EA) was gradually increased *via* transesterification in polymerization solution, whereas the MA content slowly decreased by participating in polymerization and transesterification.

Particularly, the rate of polymerization and transesterification depended on the amount of Al and the reaction temperature (Table 2 and Fig. S5†). However, in the case of [Al]_0_ = 40 mM, although the polymerization rate was much faster than the other cases, the initial transesterification rate remained slow, indicating unfavorable to poor control of gradient sequence in the polymer chain.

**Table 2:** Effect of Al(acac)_3_ on the concurrent tandem LRP of MA with EtOH^a^

Entry	[Al]_0_ (mM)	Temp. (°C)	Time (h)	Conv.^b^ (%)	*M*_n_ c	*M*_w_/*M*_n_^c^	*F*_cum,MA_/*F*_cum,EA_^b^ (%/%)
1	10	80	48	66	12 400	1.43	89/11
2	20	40	48	52	10 000	1.29	97/3
3	20	60	48	69	13 400	1.24	93/7
4	20	80	48	82	14 600	1.36	89/11
5	40	80	48	84	10 900	1.36	92/8

a [MA]_0_ = 2.0 M; [EBP]_0_ = 20 mM; [RuCp*]_0_ = 10 mM in toluene/EtOH (1/1, v/v) at 80 °C.

b Conversion was determined by ^1^H NMR.

c Measured by size-exclusion chromatography calibrated with PMMA standards in THF (40 °C, flow rate 0.3 mL min^−1^).

However, the function of cumulative (*F*_cum_) contents and instantaneous (*F*_inst_) contents were good tools for the explaining the composition of gradient sequences in polymer chains.^22,27^ To evaluate the well-controlled gradient sequence in the polymer chain, the *F*_cum_ in isolated polymer chains was calculated using ^1^H NMR (Fig. 3 (right) and Fig. S6–S8†). While the *F*_cum,MA_ (solid line) gradually decreased, *F*_cum,EA_ gradually increased from 0 to 11% in the obtained polymer chains. These results indicated that EA generated by transesterification gradually participated in the polymerization; thus, the content of EA in the polymer chain increased. In addition, *F*_inst,EA_ (dash line), which represents the differential increasing of *F*_cum,EA_, also gradually increased, with the increasing of the total conversion of monomers from 0 to 67% indicating that the gradient copolymer was successfully obtained *via* concurrent tandem LRP. The fairly controlled molecular weight and the narrow MWDs of obtained polymers were observed by SEC analyses (Table 2 and Fig. S9†).

**Fig. 4:**
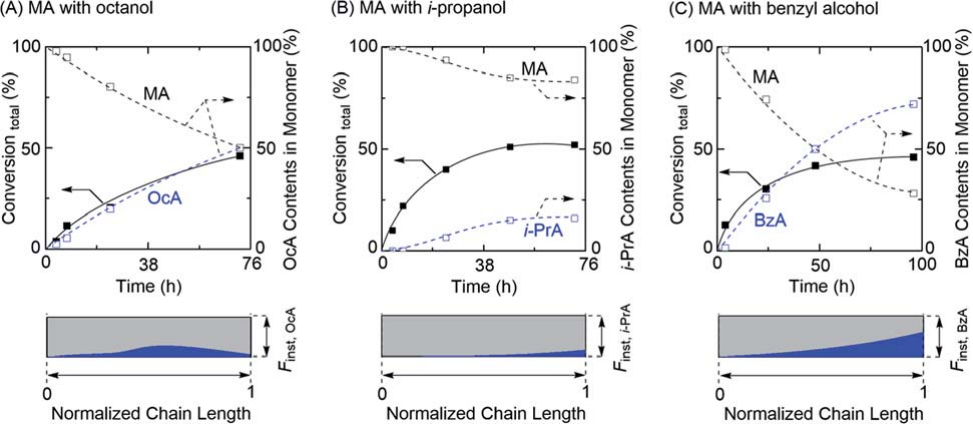
Gradient copolymerization of MA (top) with (A) octanol, (B) *iso*-propanol, and (C) benzyl alcohol *via* concurrent tandem LRP and monomer compositions *via F*_inst,RA_ in normalized polymer chain (bottom): [MA]_0_ = 2.0 M; [EBP]_0_ = 20 mM; [RuCp*]_0_ = 10 mM; [Al(acac)_3_]_0_ = 20 mM in toluene/alcohols (1/1, v/v) at 80 °C.

Encouraged by the successful gradient copolymerization of MA with EtOH, the copolymerizations of MA with varying alcohols such as octanol (long alkyl chain), *iso*-propanol (secondary), and benzyl alcohol were further explored (Fig. 4, Table 3, and Fig. S10–S12†). The MA and the RA newly generated by transesterification in concurrent tandem catalysis participated in polymerization and gave a similar monomer composition pattern in obtained polymer chains. In particular, the well-controlled gradient sequence of BzA in the obtained polymer chain was observed for the polymerization with benzyl alcohol while the polymerization with octanol showed a less controlled gradient sequence of OcA in the product. This likely occurred owing to a long alkyl chain, which might have affected the transesterification efficiency. However, unfortunately, the total monomer conversions of those polymerizations did not reach a high conversion and especially, the polymerization with *iso*-propanol showed low transesterification efficiency, which lead to a low content of *i*-PrA in the gradient sequence. It can be owing to the lower reactivity of transesterification for the secondary alcohol than that of primary alcohol. Regardless, concurrent tandem polymerization system using Al(acac)_3_ as an additive for the LRP and as a catalyst for transesterification of the acrylate monomer with varying alcohols provides acrylate-based gradient copolymers, despite the remaining challenges regarding well-controlled gradient sequences in the polymer chain as well as molecular weight control.

**Table 3:** Gradient copolymerization of MA with varying alcohols *via* concurrent tandem LRP^a^

Entry	Alcohols	Time (h)	Conv.^b^ (%)	*M*_n_ c	*M*_w_/*M*_n_^c^	*F*_cum,MA_/*F*_cum,EA_^b^ (%/%)
1	Ethanol	48	82	14 600	1.36	89/11
2	Octanol	72	50	14 000	1.19	92/8
3	*iso*-Propanol	72	52	8000	1.34	92/8
4	Benzyl alcohol	96	46	14 000	1.27	86/24

a [MA]_0_ = 2.0 M; [EBP]_0_ = 20 mM; [RuCp*]_0_ = 10 mM; [Al(acac)_3_]_0_ = 20 mM in toluene/alcohols (1/1, v/v) at 80 °C.

b Conversion was determined by ^1^H NMR.

c Measured by size-exclusion chromatography calibrated with PMMA standards in THF (40 °C, flow rate 0.3 mL min^−1^).

**Fig. 5:**
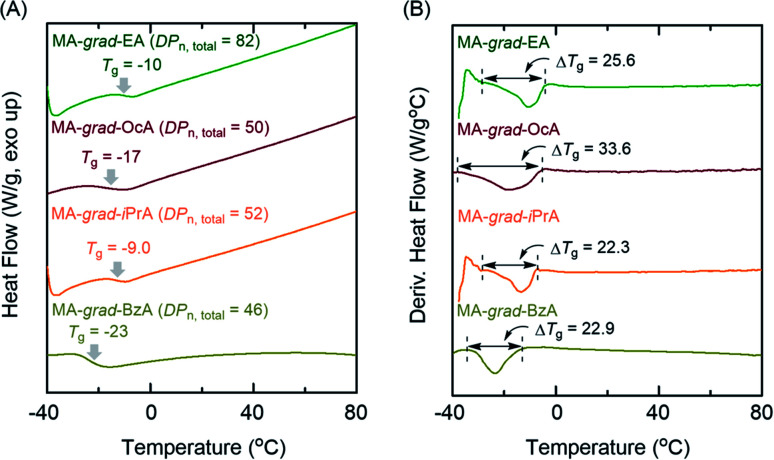
Extended DSC thermograms (2^nd^ heating process at 10 °C min^−1^ after heating up to 120 °C) of (A) the obtained gradient copolymers and (B) their differentiated DSC thermograms.

We then evaluated the effects of the gradient sequence in the polymer chain on the glass transition behaviors with differential scanning calorimetry (DSC). In general, gradient copolymers have broader phase transition ranges than the corresponding each homopolymers.^7,28^ Here, the obtained gradient copolymers indicated broad glass transition temperatures (*T*_g_) ranges (Fig. 5) compared with PMA homopolymers (Δ*T*_g_ = 14.2 °C for PMA_43_ and Δ*T*_g_ = 14.5 °C for PMA_100_ in Fig. S13†). For example, MA-*grad*-OcA, where the PMA and POcA homopolymers have different *T*_g_s, showed a broad transition in differentiated DSC profiles, supporting that the control of the gradient sequence in the polymer chain successfully occurred (Δ*T*_g_ = 33.6 °C). In addition, other series of gradient copolymers exhibited a similarly broader glass transition region, with Δ*T*_g_ = 22–25 °C, than PMA homopolymers, which would be attributed to their gradient sequence controlled polymer chains. These results indicate that Al(acac)_3_ cocatalyzed concurrent tandem polymerization of MA can successfully provide the acrylate-based gradient copolymers.

## Conclusions

Al(acac)_3_ was an effective catalyst for the transesterification of MA with various alcohols in the presence of MS 4 Å and separately, it provided good catalysis as a cocatalyst for Ru-catalyzed LRP. These two roles of Al(acac)_3_ for each reaction were successfully synchronized in concurrent tandem LRP, which led the gradient copolymers carrying well-controlled gradient sequences in the polymer chain in one-pot synthesis. Particularly, the monomer library, which was almost limited to the methyl methacrylate type monomers using metal alkoxide catalyzed concurrent tandem LRP, is expanded to the synthesis of acrylate-based gradient copolymers.

## Author contributions

The manuscript was written through the contributions of all authors. All authors have given approval to the final version of the manuscript.

## Conflicts of interest

There are no conflicts to declare.

## Supplementary Material

RA-011-D1RA04595D-s001
