# Supplementary material for: One-pot synthesis for gradient copolymers via concurrent tandem living radical polymerization: mild and selective transesterification of methyl acrylate through Al(acac)3 with common alcohols
Source: RSC Adv. 2021 Jul 28;11(42):26049–55. doi: 10.1039/d1ra04595d (PMC9037116; doi:10.1039/d1ra04595d)
Supplement: RA-011-D1RA04595D-s001 [file RA-011-D1RA04595D-s001.pdf]

Electronic Supplementary Material (ESI) for RSC Advances.
This journal is © The Royal Society of Chemistry 2021

Supporting Information

One-Pot Synthesis for Gradient Copolymers via Concurrent Tandem Living Radical Polymerization: Mild and Selective Transesterification of Methyl Acrylate through $Al(acac)_3$ with Common Alcohols

Tam Thi-Thanh Huynh,[1,2] Si Eun Kim,[1] Soon Cheon Kim,[1] Jin Chul Kim,[1] Young Il Park,[1] Ji-Eun Jeong,[1] Hyeonuk Yeo[2,3], and Sang-Ho Lee[1]**

[1]Center for Advanced Specialty Chemicals, Korea Research Institute of Chemical Technology, Ulsan 44412, Republic of Korea

[2]Department of Science Education, Kyungpook National University, Daegu, 41566, Republic of Korea

[3]Department of Chemistry Education and Department of Pharmacy, Kyungpook National University, Daegu, 41566, Republic of Korea

E-mail: slee@krict.re.kr; yeo@knu.ac.kr

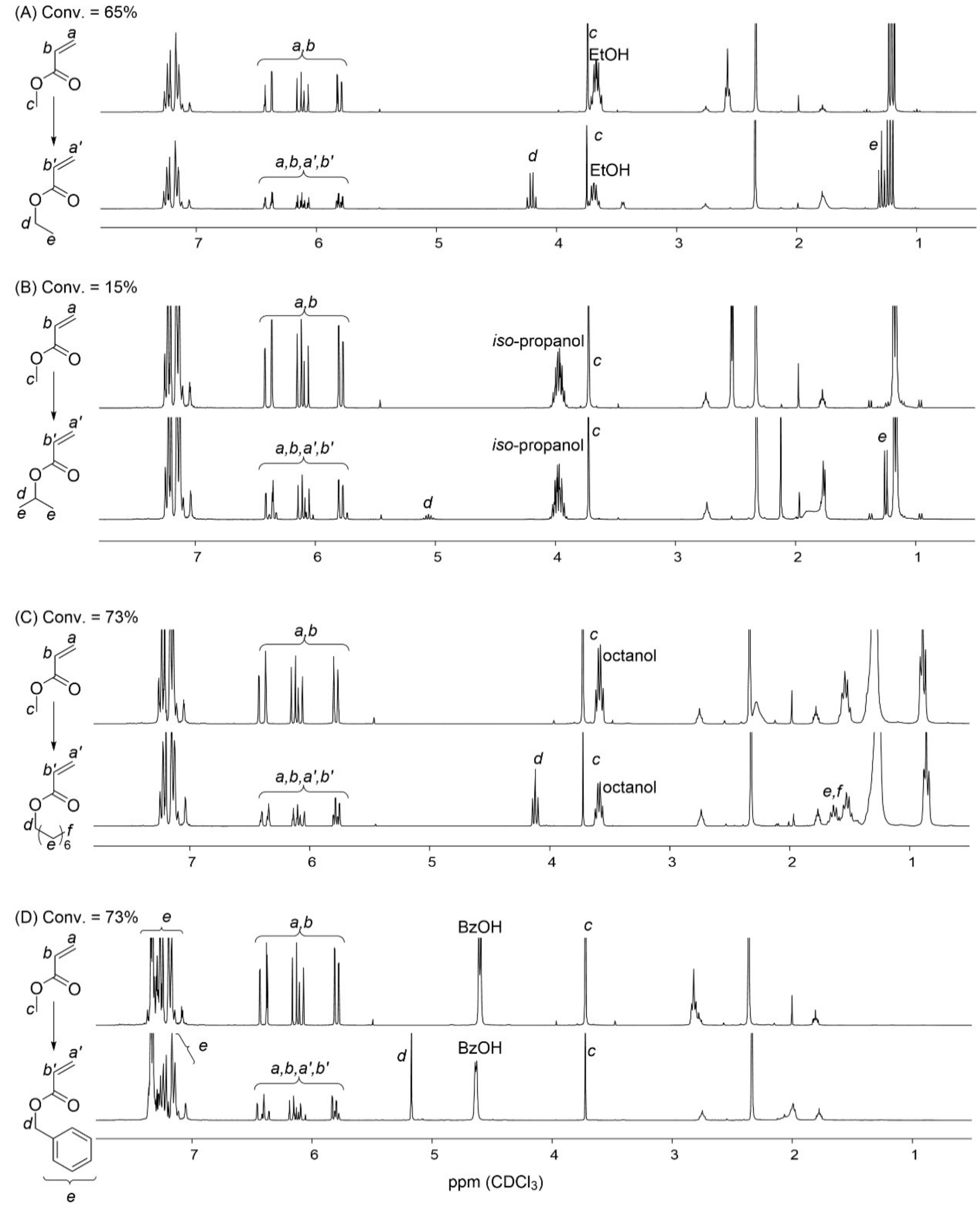

Figure S1. Al(acac)$_3$-catalyzed transesterification of MA with various alcohols: $[MA]_0$ = 2.0 M $[Al(acac)_3]_0$ = 20 mM in toluene/alcohols (1/1, v/v) at 80°C. (A) Ethanol (EtOH), (B) *iso*-propanol, (C) octanol, and (D) benzyl alcohol (BzOH).

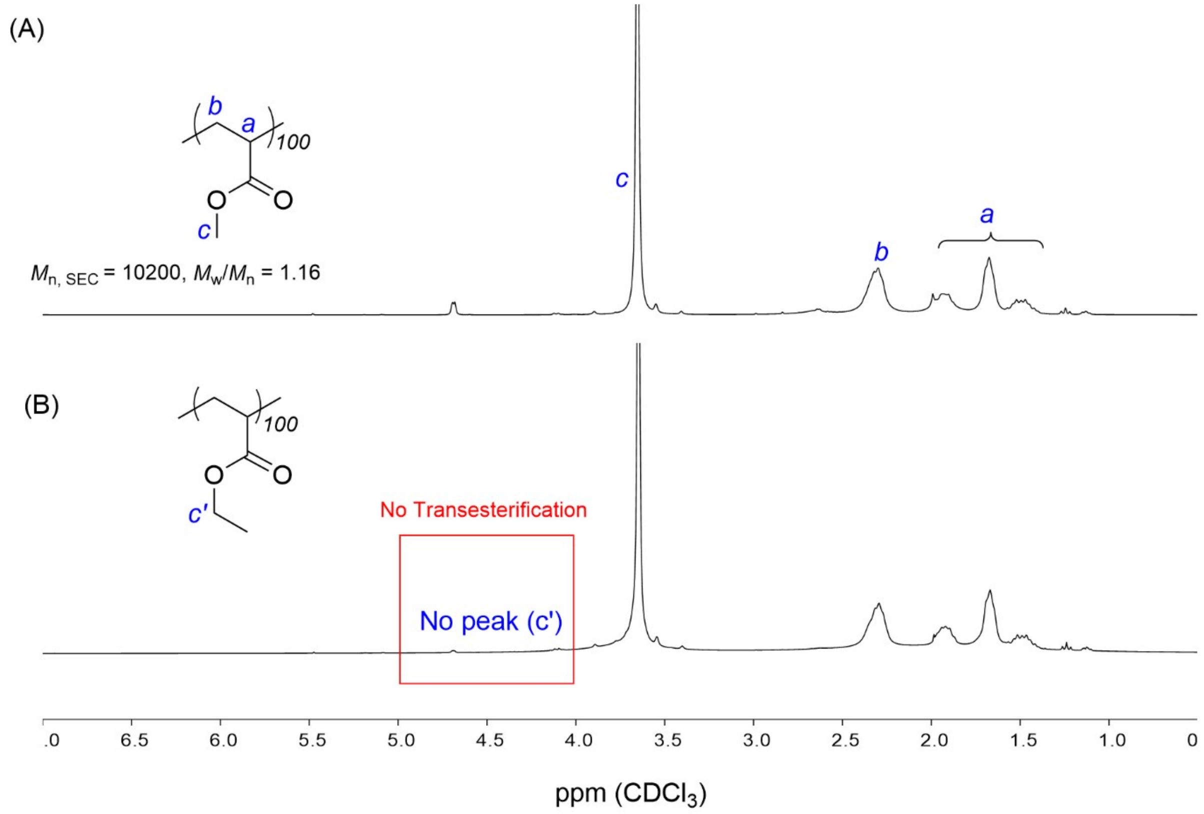

Figure S2. Al(acac)$_3$-catalyzed transesterification of [PMA ($DP_{n,\ NMR}$ = 100)]$_0$ = 20 mM; [Al(acac)$_3$]$_0$ = 20 mM in toluene/alcohols (1/1, v/v) at 80°C.

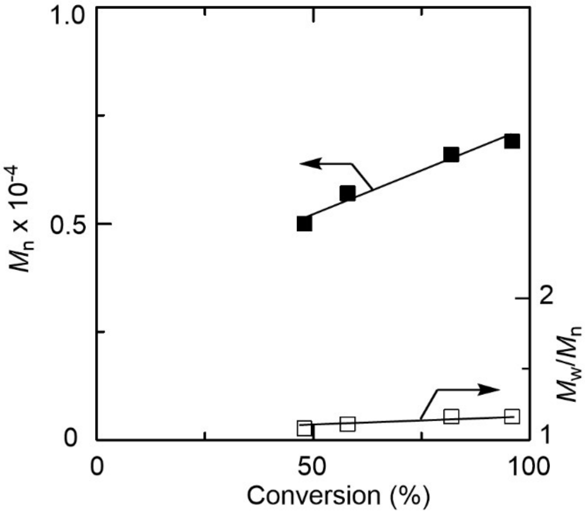

Figure S3. Control of molecular weight of poly(MA) : $[MA]_0$= 2.0 M; $[EBP]_0$ =20 mM; $[RuCp^*]_0$= 10 mM; $[Al(acac)_3]_0$= 10 mM in toluene at 80°C.

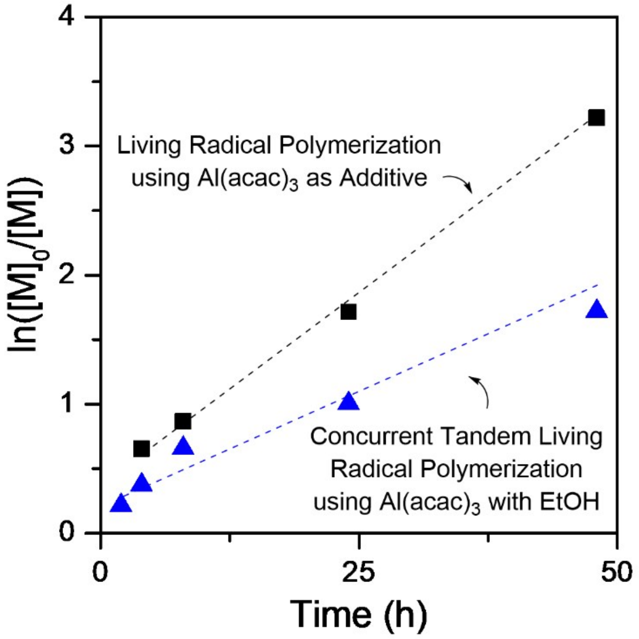

Figure S4. Kinetic plot of homolymerization of MA (black square): $[MA]_0$= 2.0 M; $[EBP]_0$ =20 mM; $[RuCp^*]_0$= 10 mM; $[Al(acac)_3]_0$= 10 mM in toluene at 80°C and gradient copolymerization of MA (blue triangle): $[MA]_0$ = 2.0 M; $[EBP]_0$ = 20 mM; $[Ru(Cp^*)Cl(PPh_3)_2]_0$ = 10 mM; $[Al(acac)_3]_0$= 20 mM in toluene/ethanol (1/1, v/v) at 80°C.

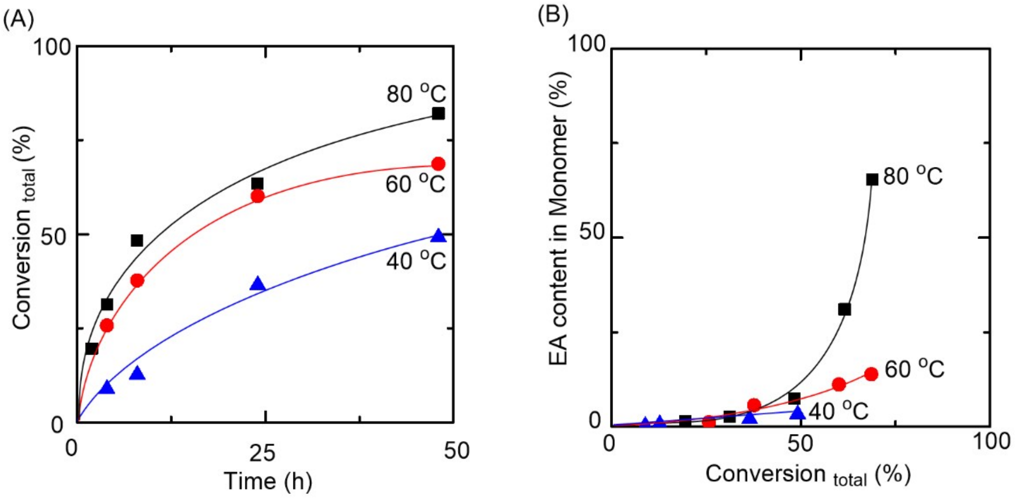

Figure S5. Effect of temperature on MA-EA gradient copolymer by concurrent tandem LRP. (A) Time-total conversion and (B) monomer content of EA in polymerization solution: $[MA]_0$ = 2.0 M; $[EBP]_0$ = 20 mM; $[Ru(Cp^*)Cl(PPh_3)_2]_0$ = 10 mM; $[Al(acac)_3]_0$= 20 mM in toluene/ethanol (1/1, v/v) at 40°C, 60°C and 80°C.

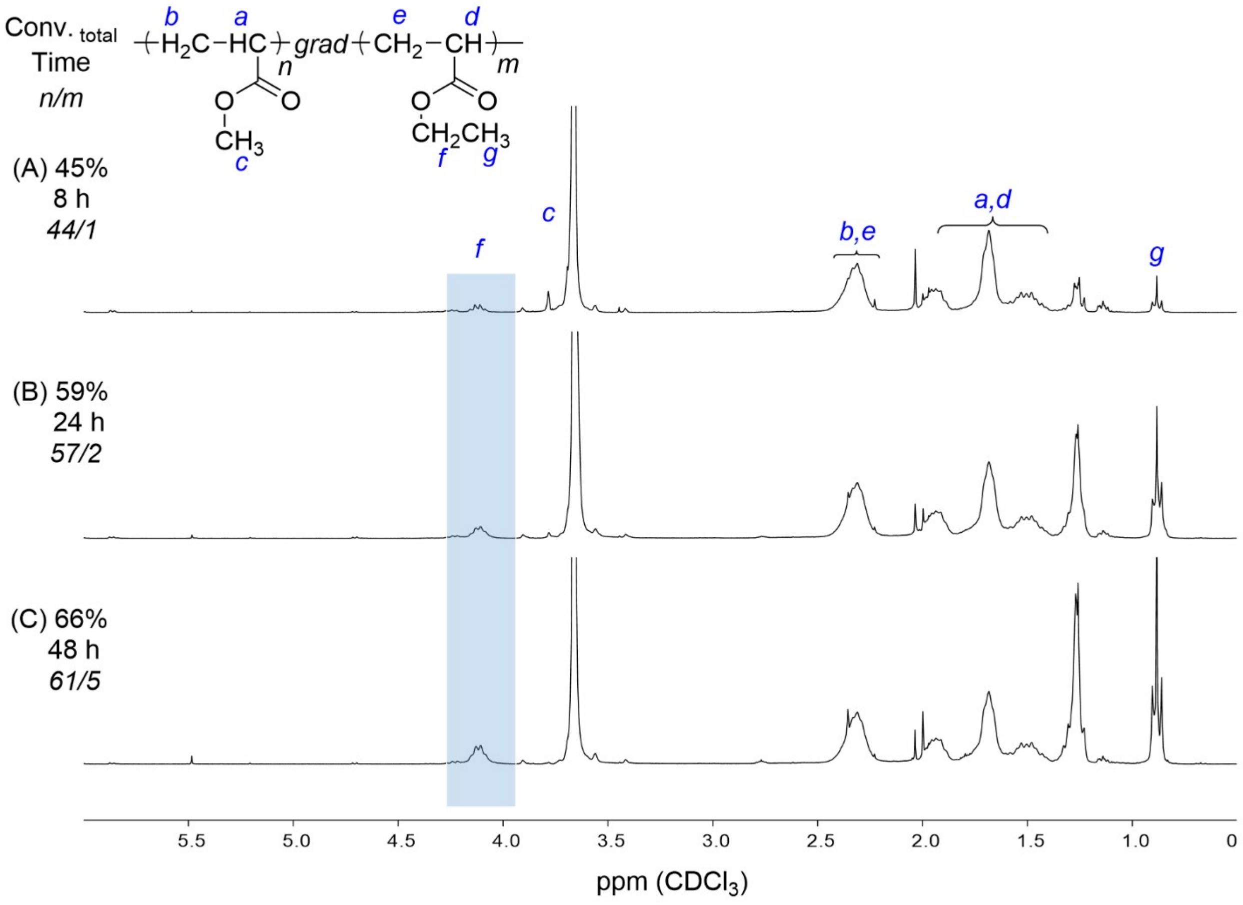

Figure S6. ^{1}H NMR spectrum of the sample obtained from the concurrent tandem living radical copolymer of MA with EtOH in toluene/EtOH (1/1): $[MA]_0$ = 2.0 M; $[EBP]_0$ = 20 mM; $[Ru(Cp^*)Cl(PPh_3)_2]_0$ = 10 mM; $[Al(acac)_3]_0$ = 10 mM at 80°C.

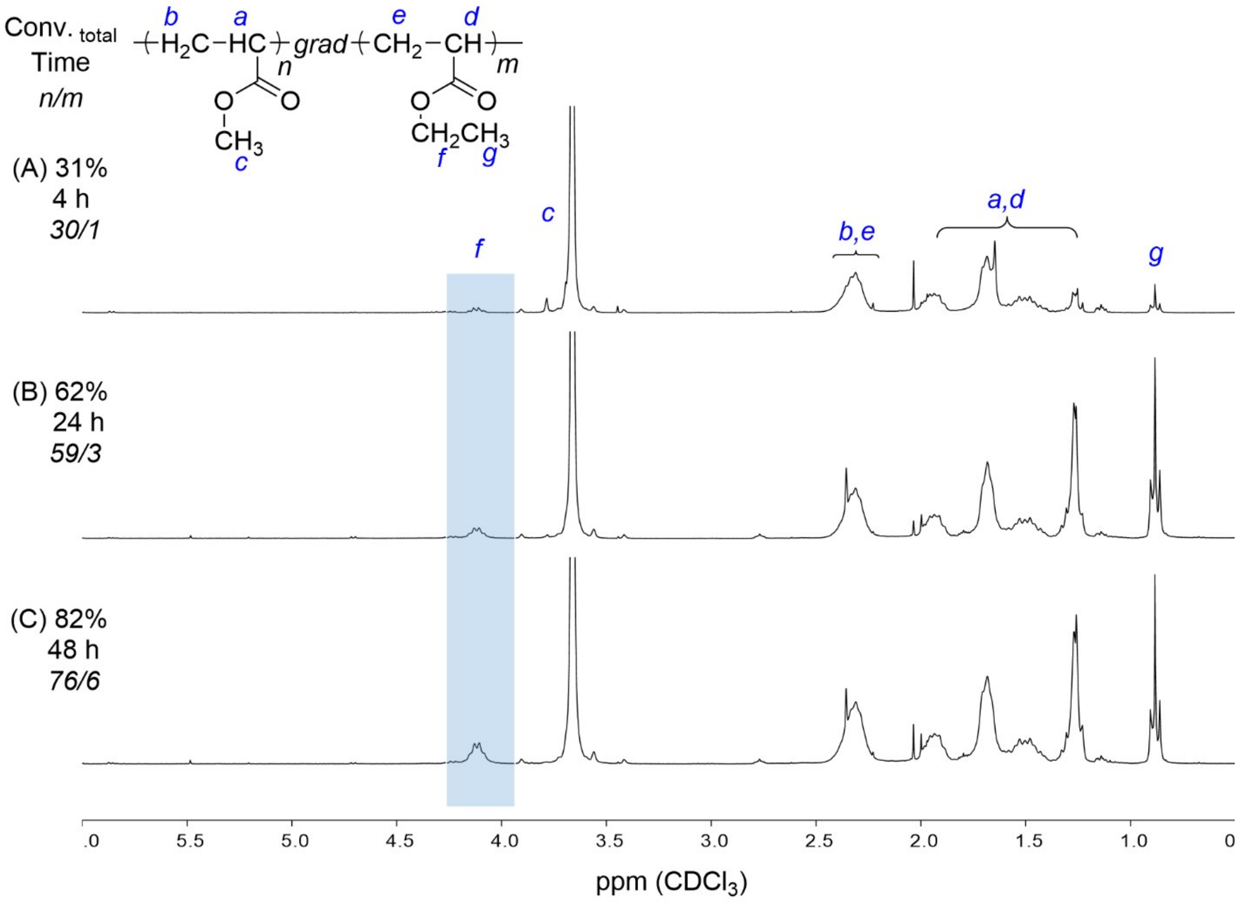

Figure S7. ^{1}H NMR spectrum of the sample obtained from the concurrent tandem living radical copolymer of MA with EtOH in toluene/EtOH (1/1): $[MA]_0$ = 2.0 M; $[EBP]_0$ = 20 mM; $[Ru(Cp^*)Cl(PPh_3)_2]_0$ = 10 mM; $[Al(acac)_3]_0$ = 10 mM at 80°C.

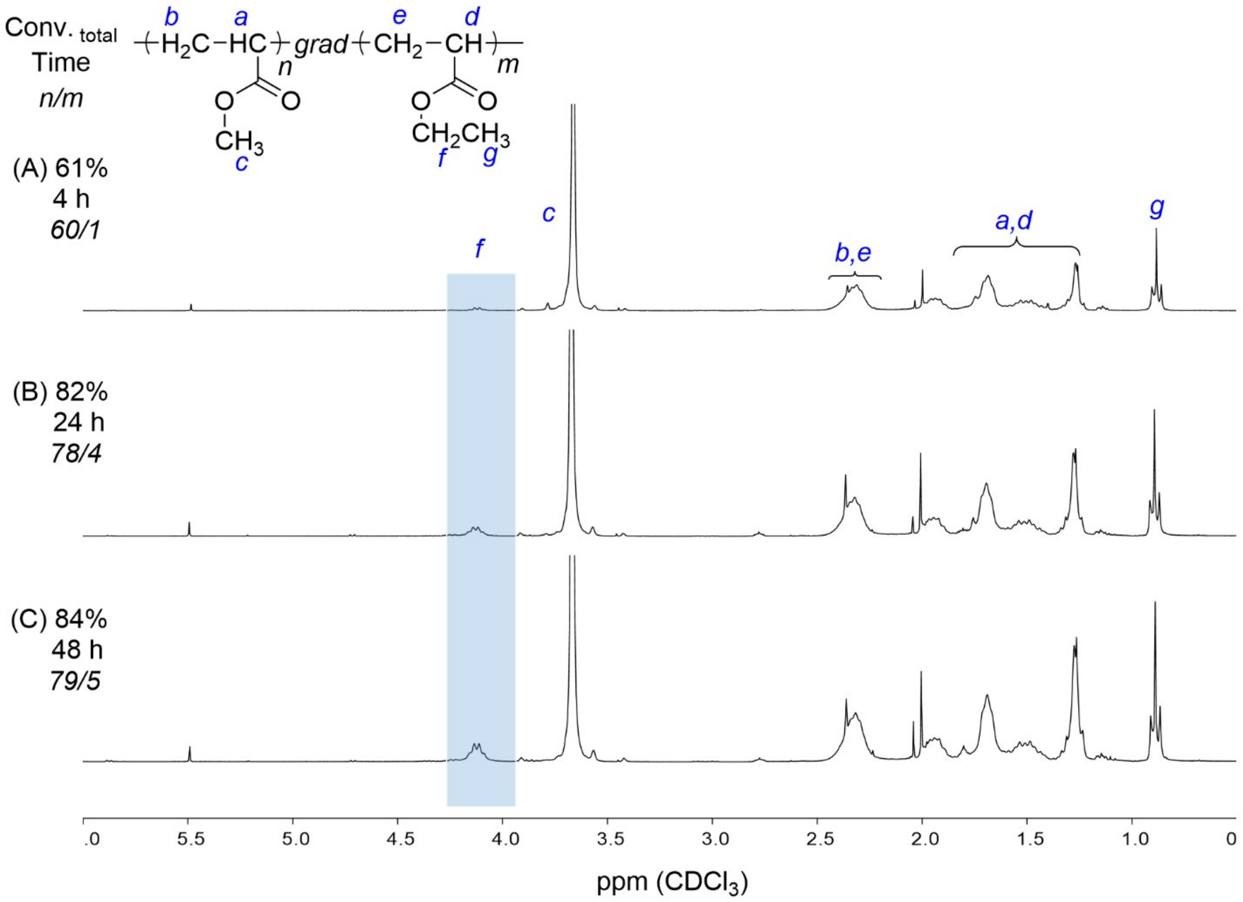

Figure S8. ^{1}H NMR spectrum of the sample obtained from the concurrent tandem living radical copolymer of MA with EtOH in toluene/EtOH (1/1): $[MA]_0$ = 2.0 M; $[EBP]_0$ = 20 mM; $[Ru(Cp^*)Cl(PPh_3)_2]_0$ = 10 mM; $[Al(acac)_3]_0$ = 40 mM at 80°C.

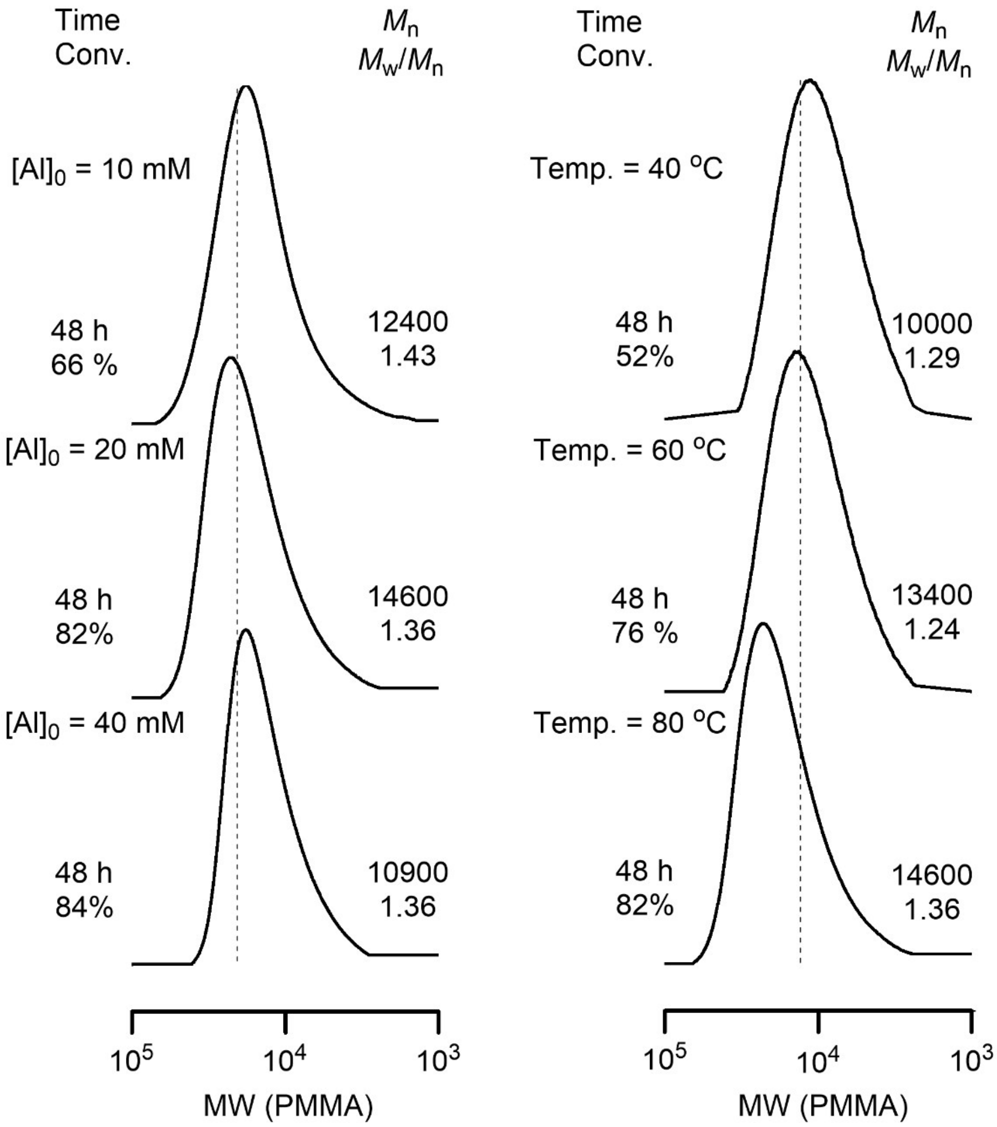

Figure S9. SEC curves of MA/EA gradient copolymer synthesis depending on the polymerization conditions.

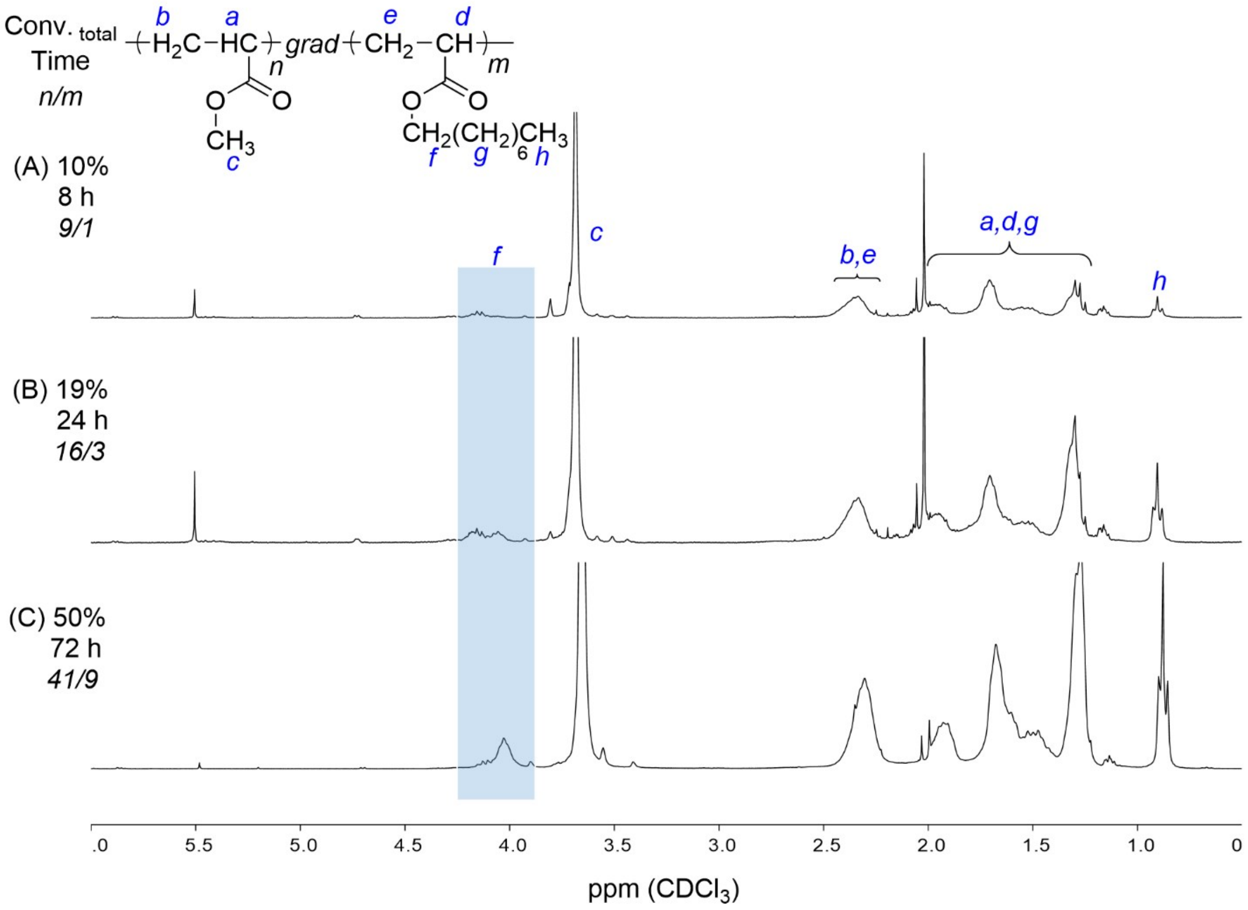

Figure S10. ^{1}H NMR spectrum of the sample obtained from the concurrent tandem living radical copolymer of MA with octanol in toluene/octanol (1/1): $[MA]_0$ = 2.0 M; $[EBP]_0$ = 20 mM; $[Ru(Cp^*)Cl(PPh_3)_2]_0$ = 10 mM; $[Al(acac)_3]_0$ = 20 mM at 80°C.

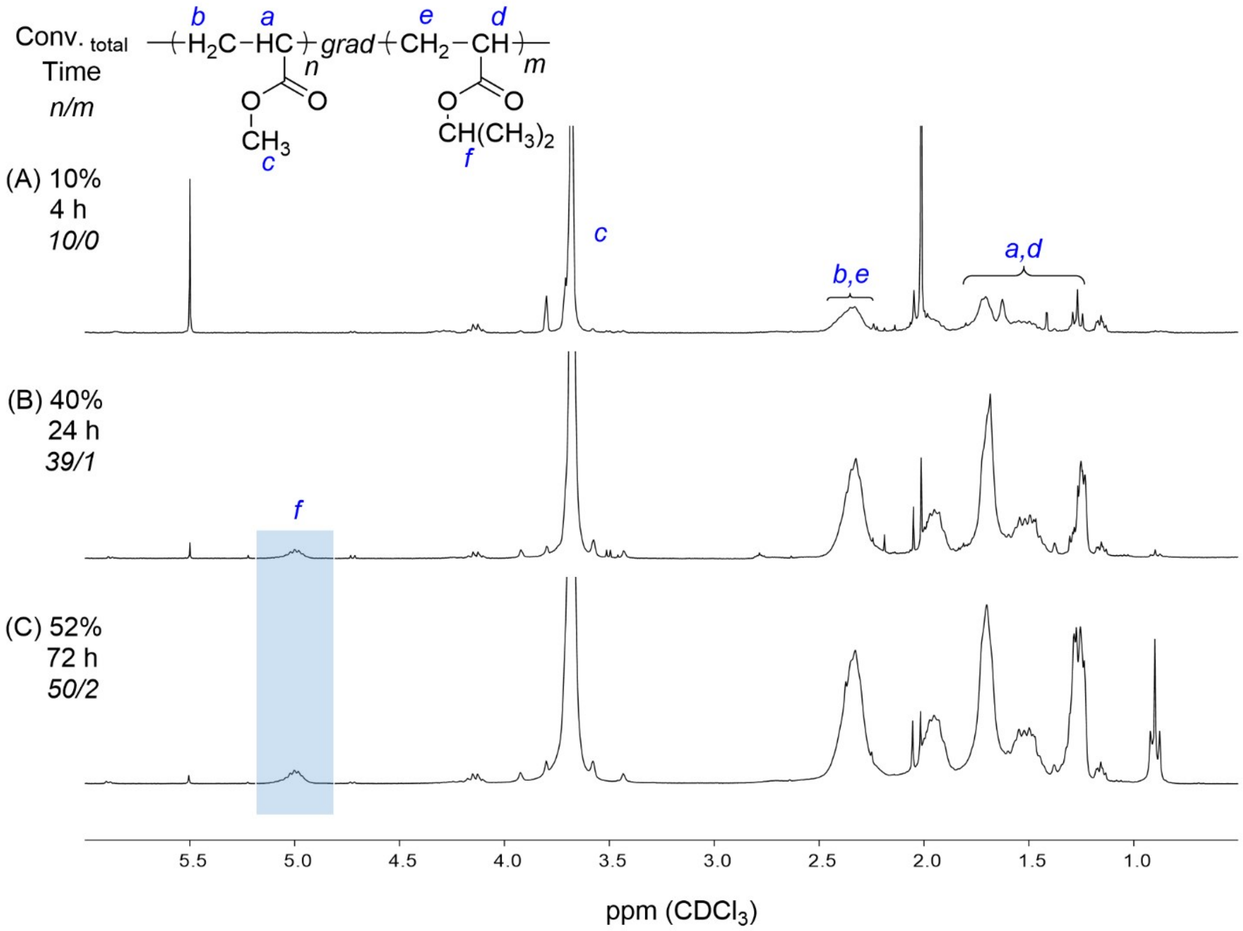

Figure S11. ^{1}H NMR spectrum of the sample obtained from the concurrent tandem living radical copolymer of MA with *iso*-propanol in toluene/*iso*-propanol (1/1): $[MA]_0$ = 2.0 M; $[EBP]_0$ = 20 mM; $[Ru(Cp^*)Cl(PPh_3)_2]_0$ = 10 mM; $[Al(acac)_3]_0$ = 20 mM at 80°C.

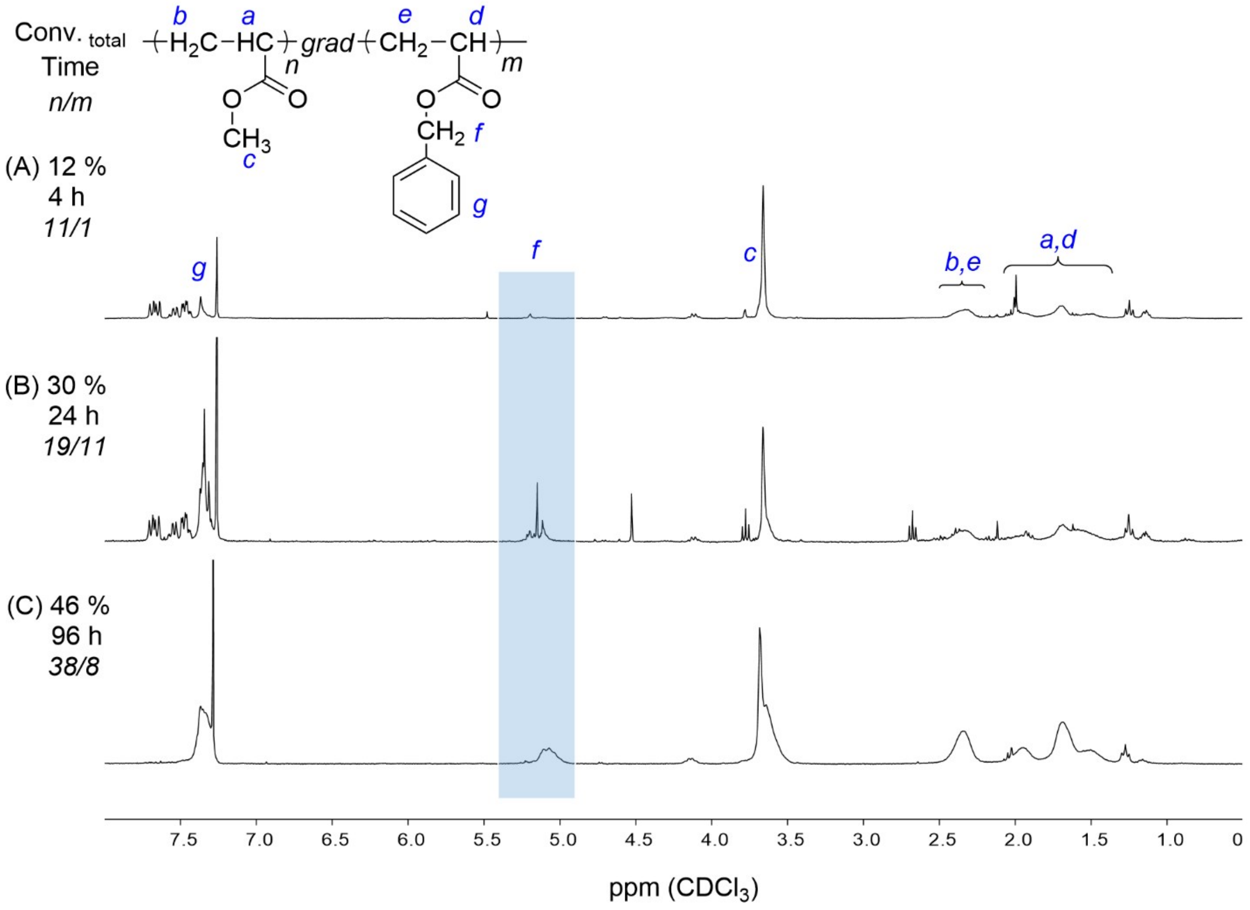

Figure S12. ^{1}H NMR spectrum of the sample obtained from the concurrent tandem living radical copolymer of MA with benzyl alcohol in toluene/benzyl acohol (1/1, v/v): $[MA]_0$ = 2.0 M; $[EBP]_0$ = 20 mM; $[Ru(Cp^*)Cl(PPh_3)_2]_0$ = 10 mM; $[Al(acac)_3]_0$ = 20 mM at 80°C.

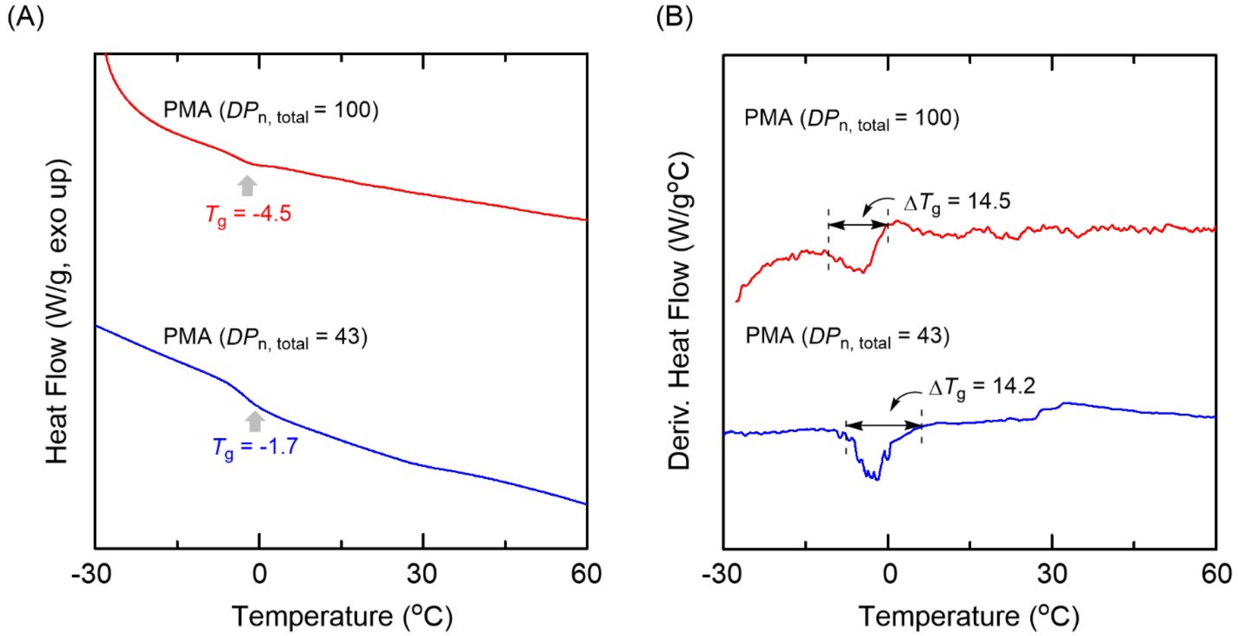

Figure S13. Extended DSC thermograms (2nd heating process at 10 °C min^{-1} after heating up to 100 °C) of (A) homopolymers and (B) their differentiated DSC thermograms.
